# Unburdening dementia – a basic social process grounded theory based on a primary care physician survey from 25 countries

**DOI:** 10.1080/02813432.2020.1794166

**Published:** 2020-07-28

**Authors:** Ferdinando Petrazzuoli, Shlomo Vinker, Sebastian Palmqvist, Patrik Midlöv, Jan De Lepeleire, Alessandro Pirani, Thomas Frese, Nicola Buono, Jette Ahrensberg, Radost Asenova, Quintí Foguet Boreu, Gülsen Ceyhun Peker, Claire Collins, Miro Hanževački, Kathryn Hoffmann, Claudia Iftode, Tuomas H. Koskela, Donata Kurpas, Jean Yves Le Reste, Bjørn Lichtwarck, Davorina Petek, Diego Schrans, Jean Karl Soler, Sven Streit, Athina Tatsioni, Péter Torzsa, Pemra C. Unalan, Harm van Marwijk, Hans Thulesius

**Affiliations:** a Center for Primary Health Care Research, Department of Clinical Sciences in Malmö, Lund University, Malmö, Sweden; b Department of Family Medicine, Sackler Faculty of Medicine, Tel Aviv University, Tel Aviv, Israel; c Clinical Memory Research Unit, Department of Clinical Sciences in Malmö, Lund University, Sweden; d Department of Public Health and Primary Care, General Practice, University of Leuven, Leuven, Belgium; e Family and Nursing Home Practice – Memory Clinic, Alzheimer’s Association “Francesco Mazzucca” Onlus, Ferrara, Italy; f Institute of General Practice and Family Medicine, Medical Faculty, Martin-Luther-University Halle-Wittenberg, Halle/Saale, Germany; g SNAMID (National Society of Medical Education in General Practice), Caserta, Italy; h Research Center for Emergency Medicine, Aarhus University, Aarhus, Denmark; i Department of Urology and General Medicine, Medical University of Plovdiv, Plovdiv, Bulgaria; j Institut Universitari d’Investigació en Atenció Primària- IDIAP Jordi Gol, Universitat Autònoma de Barcelona, Catalonia, Spain; k Department of Family Medicine, Ankara University School of Medicine, Ankara, Turkey; l Irish College of General Practitioners, Dublin, Ireland; m Director Health Care Center of Zagreb, Zagreb, Croatia; n Department of General Practice and Family Medicine, Center for Public Health, Medical University of Vienna, Vienna, Austria; o Timis Society of Family Medicine, Timisoara, Romania; p Department of General Practice, University of Tampere, Finland; q Family Medicine Department, Wroclaw Medical University, Wroclaw, Poland; r EA 7479 SPURBO. Department of General Practice, Université de Bretagne Occidentale, Brest, France; s The Research Centre for Age-related Functional Decline and Disease, Innlandet Hospital Trust, Ottestad, Norway; t Department of Family medicine, Faculty of Medicine, University of Ljubljana, Ljubljana, Slovenia; u Department of Family Medicine and Primary, Health Care Ghent University, Ghent, Belgium; v Mediterranean Institute of Primary Care, Attard, Malta; w Institute of Primary Health Care (BIHAM), University of Bern, Bern, Switzerland; x Department of Internal Medicine, General Medicine, Faculty of Medicine, University of Ioannina School of Health Sciences, Ioannina, Greece; y Department of Family Medicine, Faculty of Medicine, Semmelweis University, Budapest, Hungary; z Department of Family Medicine, Marmara University Medical Faculty, Istanbul, Turkey; aa Brighton and Sussex Medical School, University of Brighton, UK; ab Department of Medicine and Optometry, Linnaeus University, Kalmar, Sweden

**Keywords:** Dementia, drug prescription, primary care, unburdening, elderly people, grounded theory

## Abstract

**Objective:**

To explore dementia management from a primary care physician perspective.

**Design:**

One-page seven-item multiple choice questionnaire; free text space for every item; final narrative question of a dementia case story. Inductive explorative grounded theory analysis. Derived results in cluster analyses. Appropriateness of dementia drugs assessed by tertiary care specialist.

**Setting:**

Twenty-five European General Practice Research Network member countries.

**Subjects:**

Four hundred and forty-five key informant primary care physician respondents of which 106 presented 155 case stories.

**Main outcome measures:**

Processes and typologies of dementia management. Proportion of case stories with drug treatment and treatment according to guidelines.

**Results:**

*Unburdening* dementia – a basic social process – explained physicians’ dementia management according to a grounded theory analysis using both qualitative and quantitative data. Unburdening starts with *Recognizing* the dementia burden by *Burden Identification* and *Burden Assessment* followed by *Burden Relief*. Drugs to relieve the dementia burden were reported for 130 of 155 patients; acetylcholinesterase inhibitors or memantine treatment in 89 of 155 patients – 60% appropriate according to guidelines and 40% outside of guidelines. More Central and Northern primary care physicians were allowed to prescribe, and more were engaged in dementia management than Eastern and Mediterranean physicians according to cluster analyses. Physicians typically identified and assessed the dementia burden and then tried to relieve it, commonly by drug prescriptions, but also by community health and home help services, mentioned in more than half of the case stories.

**Conclusions:**

Primary care physician dementia management was explained by an *Unburdening* process with the goal to relieve the dementia burden, mainly by drugs often prescribed outside of guideline indications.

**Implications:** Unique data about dementia management by European primary care physicians to inform appropriate stakeholders.

**Key points**
Dementia as a syndrome of cognitive and functional decline and behavioural and psychological symptoms causes a tremendous burden on patients, their families, and society.
•We found that a basic social process of *Unburdening* dementia explained dementia management according to case stories and survey comments from primary care physicians in 25 countries.
•First, *Burden Recognition* by *Identification* and *Assessment* and then *Burden Relief* – often by drugs.
•Prescribing physicians repeatedly broadened guideline indications for dementia drugs. The more physicians were allowed to prescribe dementia drugs, the more they were responsible for the dementia work-up.
Our study provides unique data about dementia management in European primary care for the benefit of national and international stakeholders.

## Introduction

Dementia arises together with the accumulation of harms and disease burdens over the life course, and clusters with other endemic conditions exacerbating each other synergistically [1,2]. Multimorbidity and cardiovascular comorbidity show prevalences of 65–98% for those more than 65 years of age [3] with accompanying polypharmacy and potentially inappropiate medications adding to the burdens [4]. Dementia can be seen as a triad of impairments and burdens: cognitive, emotional, and physical [2] in a ‘frail brain’ [5]. Dementia eventually results in disabled instrumental and basic activities of daily life, neuropsychiatric issues, personality changes, social impairment and stigma [6]. These disease burdens are often long lasting and indeed affect family caregivers who often develop depression and physical illnesses while caring for their loved ones [7]. A systematic review dominated by qualitative studies of dementia management in primary care showed lack of support for patients, caregivers and primary care physicians and an attitude of ‘therapeutic nihilism’ [8]. There was also limited access to and knowledge about community services and resources and absence of interdisciplinary teams to enhance management [8]. Other main themes from the qualitative studies were ‘time constraints, financial constraints, stigma, diagnostic uncertainty, and disclosing the diagnosis’ [8]. With this gloomy review as background, we wanted to explore dementia management using an inductive approach from classic grounded theory where the basic research question is ‘what is going on?’ We wanted to know how primary care physicians in different countries treat their patients suffering from dementia, and to generate an explanatory theory of dementia management in primary care.

## Methods

### Study design

For this survey study directed to primary care physicians in 25 countries of the European General Practice Research Network (EGPRN), we developed a seven-item multiple choice questionnaire (MCQ). The design of the survey has been presented elsewhere [9,10]. At the end of every MCQ, optional free text comments could be added [10]. In the eighth and last survey item, we invited respondents to contribute anonymised case stories of dementia from their own practice.

We applied an analytic classic grounded theory approach characterised by inductive reasoning with no a priori hypotheses allowing both qualitative and quantitative data analysis [11–13].

### Setting

In the 25 countries, national key informants were identified and contacted face to face by author FP during meetings of the EGPRN and WONCA Europe conferences in 2013–2015. National key informants organised translation of the survey into their own languages and back translation to English. They also selected further key informants from different geographical and socio-economic regions within their country by convenience or snowball sampling.

### Participants and data

445 key informant primary care physicians from 25 countries, of which 106 physicians from 23 countries presented 155 case stories.

Data coded for this study:

- Secondary analysis of MCQ data provided by 445 key informants and 8,000 words of free text data from the same survey [9,10].
- Data from 106  key informants who provided 155 case stories consisting of 40,000 words.

### Grounded theory analysis

Classic grounded theory is the world’s most cited behavioral research method with 124,055 Google Scholar citations (15 July 2020) [11]. In classic grounded theory, hypotheses are inductively generated to explain how participants resolve their main concern abstract of time, place and people [11,13,14]. Classic grounded theory differs from other methods using only qualitative data by emphasising explanatory concepts rather than descriptions.

Theoretical memos, in the shapes of text, diagrams, and figures, were written, typed, or drawn to create a 200-page memo bank from which this paper was written. ‘Memos are the theorizing write-up of ideas about substantive codes and their theoretically coded relationships as they emerge during coding, collecting and analysing data’ [14]. These memos were analysed based on grounded theory quality principles of fit, relevance, workability and modifiability [14,16]. Then followed selective coding where the core concept guided the analysis during theoretical memoing. We coded case stories and survey text comments asking for each incident in the data ‘What is the participants’ main concern and how is it being resolved?’ and ‘What are the participants doing to resolve their main concern’ and ‘What is this a study about?’. Answers to these coding questions yielded several hundred indicators of preliminary dementia discovery, diagnosis, work up and treatment. Theoretical memos included codes related to care and caregivers – spouse, children, institutional and non-institutional care etc. We further analysed survey free text including descriptions of formal and informal rules of dementia work up and diagnostic and treatment structures of the jurisdiction in which the case story took place [9].

The core concept was theoretically coded using a basic social process [11,14–16] to explain the ongoing resolution of the main concern in the case stories.

We also ran numerous statistical analyses based on both grounded theory codes and results of MCQ questions. These analyses were included in memos to deductively fit the emerging concepts by applying the ‘constant comparative method’ of grounded theory and indeed using its dictum ‘all is data’ [14,16].

The grounded theory analysis was mainly done by the first and last author in collaboration and analytic consensus was reached during the write-up process lasting several years and ending with the submission of this manuscript. Good grounded theories should indeed be modifiable when new data enters such as relevant critique from reviewers [14,16].

### Dementia analysis

Proxies for treatment appropriateness were aligned with the guidelines for dementia drug therapy and established by a tertiary care physician researcher expert on neurocognitive disorders (author SP) [17,18]. SP also assessed the specific dementia type based on the information given in the case stories in accordance with the DSM-5 [19].

### Statistical analysis

IBM SPSS Statistics for Windows, Version 22.0. (IBM, Armonk, NY USA, 2013) was used for descriptive and exploratory statistical analyses. Cluster analysis is an exploratory method used to identify structures within the data such as homogenous groups of cases if grouping and differences between dependent and independent variables is previously unknown [10,20]. We did two-step cluster analyses with variables emerging from memos. Overall goodness-of-fit of clusters had silhouette coefficients with measures of less than 0.2 classified as poor, between 0.2 and 0.5 fair, and more than 0.5 as a good solution quality. Fair or higher was considered acceptable clustering with a cut-off score at 0.3 [20]. More advanced statistical methods such as Directed Acyclical Graphs were applied to the data in another version of this study available online [10].

In summary, this study involved a mix of data and methods emphasising an inductive approach of classic grounded theory with the goal of exploring and generating a hypothesis of what was going on in the field of primary care physician dementia management.

## Results

A multitude of burdens was the main concern surrounding the care of patients with dementia and their caregivers. Patients and caregivers were overwhelmed not only by the burden of dementia but often by multimorbidity – present in 74% of the case stories – and by behavioural and psychological symptoms of dementia.

The core concept, *Unburdening* dementia, was deduced as a label explaining the resolution of the main concern.

*Unburdening* dementia is seen as a basic social process starting with *Identifying* the burden, mostly done by family members of the patient, then *Assessing* the burden by cognitive tests, especially the Mini Mental State Examination (MMSE), the most used cognitive psychometric test internationally [21]. The dementia burden is then *Recognised* by formal caregivers and eventually pursued *by Burden Relief*, often by prescription drugs in 130 of 155 (84%) of the cases but also by community health and home help services, mentioned in more than half of the case stories. The basic social process of *Unburdening* dementia is presented in Table 1 with sub-categories and different properties.

**Table 1.** The basic social process of Unburdening Dementia.*

Unburdening Dementia - a two-step Basic Social Process		
BASIC SOCIAL PROCESS STAGE:	**Recognising** burden after burden *identification* and *assessment*.	**Burden Relief**
APPLIED TO DEMENTIA CARE :	*Identifying* cognitive and mental burden by family caregiver. *Assessing* burden by psychometric tools and then **Recognising** burden by formal caregiver.	Cognitive, mental and social **Burden Relief**
CASE STORY ILLUSTRATION:	Consultation together: spouse/child report memory loss. Physician notices lack of collaboration during consultation. Assessment with Mini Mental State Examination. Physician diagnoses the patient with dementia.	**Burden Relief** by community health services, home help services and drugs

The table illustrates the basic social process of Unburdening Dementia that emerged to explain the action in the data provided by primary care physicians across EGPRN countries. *Basic social processes are grounded theory core variables that are ‘processural’ meaning that they have two or more clear emergent stages [13,14]. Descriptive incidents of community health and home help services (such as nursing home care and dementia services) appeared in more than half of the case stories as indicators of a property of Burden Relief.

*Unburdening* explains what many physicians – both primary care physicians and secondary care specialists – were doing to help patients and their families. *Unburdening* often resulted in *Burden Relief* consisting of drug prescriptions – the most available therapeutic tool. Acetylcholinesterase inhibitors or memantine treatment was reported in 89 of 155 patients (57%): 60% of the prescriptions were appropriate according to guidelines and 40% prescribed outside of guidelines. Antidepressants were mentioned in 27% of the patients, and to a lesser degree other drugs such as antipsychotics. Only 22 out of 155 case stories did not mention any drug to relieve the dementia burden. More than two-thirds of patients in our study that were prescribed acetylcholinesterase inhibitors and/or memantine had too many exclusion criteria to be accepted into dementia drug trials [10].

In five out of 25 countries, primary care physicians were entitled to officially prescribe reimbursable dementia drugs. In many other countries, primary care physicians could diagnose dementia unofficially and prescribe specific dementia drugs to patients that would not be reimbursed by their health insurance [9,10].

The participating countries were split into types according to the ability of primary care physicians to prescribe a dementia drug which could subsequently be reimbursed by the health insurance system: ‘permissive’ = primary care physicians always able to prescribe, ‘partially permissive’ = need for first prescription by secondary care specialist, and ‘non-permissive’ = mandatory secondary care prescribing and follow-up. Some primary care physicians from ‘non-permissive’ countries prescribed dementia drugs to their patients despite the restrictions for the purpose of mercy and equity as an unburdening action.

The MCQ analysis showed that the more primary care physicians were officially allowed to prescribe dementia drugs, the more they felt responsible for the diagnostic work-up. But they also then increasingly seemed to engage multiprofessional community health and home help services in unburdening tasks, thus providing psychosocial support.

Demographics, dementia prevalence, grades of dementia drug prescribing permissiveness in the participating countries and share of dementia drugs prescribed in the case stories per region are shown in Table 2.

**Table 2.** Demographics, dementia prevalence, grades of dementia drug prescribing permissiveness in the participating countries and share of dementia drugs prescribed in the case stories per region.

Country	Population million people	Population 65 years old or over (%)	Dementia prevalence (%)	Primary care physician allowed to prescribe dementia drugs?	Number of case stories (% of total)	Proportion of typical dementia drug treatment per case per region
Denmark	5.8	18.2	1.5	PARTIAL	3 (2)	
Finland	5.5	19.4	1.7	NO	4 (3)	
Norway	5.2	15.9	1.6	YES	6 (4)	
Sweden	9.7	19.4	1.8	YES	8 (5)	
North Countries, total					21	57%
Austria	8.6	18.3	1.7	NO	3 (2)	
Belgium	11.3	17.8	1.8	PARTIAL	2 (1)	
France	66.4	18.0	1.8	PARTIAL	5 (3)	
Germany	81.2	20.8	1.9	YES	5 (3)	
Ireland	4.6	12.6	1.1	YES	35 (23)	
Switzerland	8.2	17.6	1.7	YES	5 (3)	
The Netherlands	16.9	17.3	1.5	PARTIAL	5 (3)	
West Countries, total					60	67%
Bulgaria	7.2	19.6	1.5	NO	5 (3)	
Croatia	4.2	18.4	1.5	NO	3 (2)	
Hungary	9.8	17.5	1.5	NO	3 (2)	
Poland	38.0	14.9	1.3	NO	20 (13)	
Romania	19.9	16.5	1.3	NO	10 (6)	
Slovenia	2.1	17.5	1.6	PARTIAL	4 (3)	
East Countries, total					45	40%
Greece	10.8	20.5	1.8	NO	4 (3)	
Israel	8.5	10.3	1.1	NO	7 (5)	
Italy	60.6	21.4	2.1	NO	8 (5)	
Malta	0.4	17.9	1.3	NO	1 (1)	
Spain	46.4	18.1	1.8	NO	6 (4)	
Turkey	77.7	7.7	0.4	NO	3 (2)	
Mediterranean Countries, total					29	66%
Total					155	57%

Legend: Demography, dementia prevalence, prescription rules for primary care physicians, dementia cases stories and proportion of typical dementia drug treatment from 25 countries in the EGPRN. Data on dementia prevalence by Prince et al. 2013 and on dementia drug prescribing rules by the Alzheimer Europe Association, 2012.

Statistical analytical procedures also showed the relevance of *Unburdening*. Thus, statistical data were used in memos to test the basic social process core variable. This means that the statistics supported theory generation by making it less dependent on qualitative data only, due to the large amount of data readily coded quantitatively. After basic descriptive statistics, we employed the analytic procedures below during the selective coding phase.

### (a) Cluster analyses

A two-step cluster analysis divided 151 case stories into two groups (four cases had missing data). Group 1 had 76 stories and group 2 had 75 stories. ‘Permissiveness to prescribe dementia drugs’ and ‘country’ predicted group affiliation (Figure 1).

**Figure 1. Importance of predictors of primary care physician involvement in dementia work-up and treatment. Degree of involvement is dichotomised into two groups by a two-step cluster analysis.**
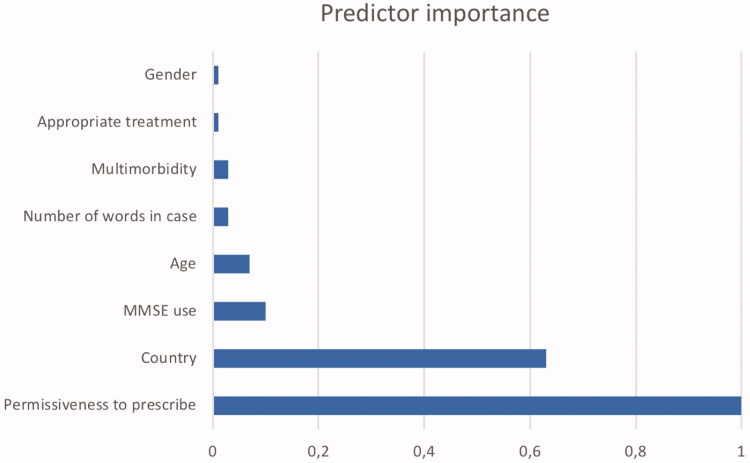
Cluster analysis is an *exploratory method* to identify structures within the data such as homogenous groups of cases if grouping is not previously known. ‘Exploratory’ means that it makes no distinction between dependent and independent variables. We used the SPSS Version 22 *two-step cluster analysis*. The most important predicting variables to allocate the informants in the two groups were ‘permissiveness to prescribe dementia drugs’ (Predictor importance PI = 1) and ‘country’ (PI = 0.61). All the other variables had a PI of < 0.02. Permissiveness: official permissiveness to prescribe typical dementia drug reimbursed by the local health care system. Country: country of the case and his/her primary care physician. Appropriate treatment: appropriate treatment according to the dementia guidelines and the label indication. Age: age of the case in years. Gender: gender of the case. MMSE: mini mental state examination. Number of words in case: word count in the case description.

Group 1 had high involvement of primary care physicians in the dementia work up and treatment. Patients were from Belgium, Denmark, Finland, France, Germany, Ireland, the Netherlands, Norway, Sweden, and Switzerland.

Group 2 had low involvement of primary care physicians in the dementia work up and treatment. Patients were from Austria, Bulgaria, Croatia, Greece, Hungary, Israel, Italy, Malta, Poland, Romania, Slovenia, Spain, and Turkey.

The different types of dementia (neurocognitive disorders) as assessed by a tertiary care physician (author SP) are shown in Table 3. Alzheimer’s disease was the most common dementia subtype followed by vascular dementia. In a third of the patients, no subtype could be specified due to lack of diagnostic information, which resulted in the unspecified diagnoses of mild or major neurocognitive disorders.

**Table 3.** Dementia drug treatment stratified by diagnostic group.

	Dementia Drug		Total number of patients
Diagnosis	No regular dementia drug	Regular dementia drug (acetylcholinesterase inhibitors/memantine)	
No diagnostic information available	4	0	4
Alzheimer’s Disease/Major Neurocognitive Disorder (*)	9	31	40
Vascular Dementia /Major Neurocognitive Disorder (†)	12	4	16
Major Neurocognitive Disorder unspecified (β)	19	31	50
Normal Pressure Hydrocephalus	1	0	1
Mild Neurocognitive Disorder unspecified	5	0	5
Major Neurocognitive Disorders, mixed aetiologies	10	22	32
Fronto-Temporal Dementia (β)	3	1	4
Dementia of Lewy Body	1	0	1
Alzheimer’s Disease/ Mild Neurocognitive Disorders	2	0	2
Total	66	89	155

Legend: Memantine and acetylcholinesterase inhibitors were considered appropriately used if they were prescribed to patients with Alzheimer’s disease (AD), mixed dementia (if AD was considered one of the components), dementia with Lewy bodies or Parkinson’s disease dementia. The indication for the treatment was considered correct regardless of dementia stage (i.e. memantine was not restricted to moderate to severe AD and acetylcholinesterase inhibitors was not restricted to mild to moderate AD) [21].

(*) = appropriate prescription of acetylcholinesterase inhibitors/memantine.

(β) = questionable prescription of acetylcholinesterase inhibitors/memantine.

(†) = inappropriate prescription of acetylcholinesterase inhibitors/memantine.

### (b) Cross-sectional associations by chi-square testing

There was a significant positive association between permissiveness to prescribe dementia drugs and primary care physicians’ diagnosis of dementia. MMSE testing was reported in 128 out of 155 cases (83%) and was significantly associated with permissiveness to prescribe dementia drugs but not with effective prescription of dementia drugs. We found no significant association between permissiveness to prescribe dementia drugs and the mentioning of dementia drugs or appropriateness of treatment. Neither did we find any significant association between appropriateness of treatment and early specialist involvement or referral. Statistical measures of the testing above are available online together with results of more advanced statistical methods applied to the data in a previous version of this study [10].

## Discussion

*Unburdening* dementia emerged as an explanation of what physicians from 25 countries did to help patients with dementia and their caregivers after analysing both qualitative and quantitative data. *Unburdening* dementia is, according to grounded theory taxonomy, a core variable and was coded as a basic social process [14–16] starting with *Recognising* the burden by *Burden Identification* and *Assessment* followed by *Burden Relief.* As a core variable, *Unburdening* is suggested as a cohesive explanation for the resolution of a main concern – the comprehensive burden of dementia – and as such a tentative explanatory theory of dementia management in primary care.

We analysed case histories and survey responses from primary care physicians in 25 countries and found that only in 14% of 155 case stories was there no mention of any drug to relieve dementia problems. In more than half of the case stories, acetylcholinesterase inhibitors and/or memantine were mentioned as being prescribed and in one quarter of case stories, guideline indications of dementia drug treatment were broadened [17,18]. The physicians also used other drugs to unburden dementia such as antidepressants.

Free text data and MCQ responses, confirmed by a two-step cluster analysis, showed that the more the primary care physicians were officially allowed to prescribe dementia drugs the more they were responsible for the dementia work-up [9,10] also engaging community health and home help services in unburdening tasks. Pro-active dementia management by primary care physicians was related to geographical region with Central and Northern European physicians showing high permissiveness to prescribe dementia drugs in comparison with a low prescribing permissiveness in Eastern European and Mediterranean countries. This finding of regional differences within Europe confirmed a pattern seen previously [9,10].

‘Label indications’ of drugs are often limited and narrow [6,18]. Yet, both primary and secondary care physicians do not always apply a strict indication perspective. They have a patient with multiple severe conditions in front of them and try to do their best to help that patient [22]. The most common tool that many physicians use to help their patients is the prescription of drugs. Physicians inevitably tend to broaden the indications for drugs which could be beneficial even though the patient does not fulfill the eligibility criteria for prescription [23]. We call this pattern of physician behaviour *Unburdening by drugs.*

A majority of patients in our study that were prescribed specific dementia drugs would not have been accepted in dementia drug trials that sit as the base for the alleged evidence of their effects [10]. Patients in our study were indeed on average 80 years old and had significant co-morbidity. This suggests a discrepancy between real world primary care dementia patients and patients from acetylcholinesterase inhibitor trials where the efficacy of these drugs was determined. Patients in our study were thus old, with high co-morbidity and a late diagnosis often due to the difficulty of establishing a diagnosis. The discrepancy further applies to patients being included in disease modifying anti-amyloid trials aimed at relieving the dementia burden at the micro level [24,25]. Our study suggests that since many patients with dementia in primary care are treated outside of the traditional protocols for the drug trials that were designed to prove their effects, it would seem logical to expand the drug trial populations to include typical primary care patients.

### Limitations

Day care rehabilitation, respite care and caregiver support [26] etc. are much less mentioned as *Burden Relief* procedures in our study as compared to prescribing dementia drugs of sometimes marginal therapeutic effect [17,18]. Why *Unburdening by drugs* emerged as the most common *Burden Relief* strategy used by the primary care physicians could perhaps be explained by a ‘prescription reflex’ response against therapeutic nihilism [27]. The big picture of the case stories analysed for this study resembles data from a systematic review of barriers to dementia management which showed limited access to and knowledge about community services and absence of interdisciplinary teams as well as attitudes of therapeutic nihilism [8]. The respondents in our study could have been biased for the eighth case story survey item by questions 3 and 4 in the MCQ survey that asked about drug treatment.

Our recruitment and sampling of key respondents was strategic, which limits generalizability. Also, the proportion of case story data from Ireland and Poland was higher than from other countries and differences within countries are not considered since the survey was anonymous. Yet, we used the same data collection approach for all countries. Moreover, with hundreds of pages of case story text and free text responses from several hundred primary care physicians we reached conceptual saturation according to grounded theory principles of fit, relevance, workability and modifiability [14,16]. These are qualities that in traditional qualitative data analysis often are labeled trustworthiness and generalizability. Hence, we suggest that *Unburdening* as a basic social process is enough abstract of time, place and people to be considered useful for a general understanding of practice habits of primary care physicians.

Our inductive method was based on classic grounded theory that mostly analyses qualitative data. Yet, we used quantitative measures too, rare for classic grounded theory but they fit our data [11–13]. Thus, *Unburdening* dementia emerged as a relevant basic social process. Since our data was based on what informants chose to report the results should, as in all classic grounded theory research, not be seen as facts but as tentative hypotheses. This is a limitation in deductive research but fits the tenets of the inductive property of the classic grounded theory method.

In many case stories, specification of the type of dementia was not given by the respondents and this is similar to other studies in primary care where the subtype of dementia was not considered [28]. Hence, a tertiary care physician researcher carefully examined all case stories and tried to establish the specific dementia type in accordance with DSM-5 [19].

### Contribution to policy and practice

High permissiveness to prescribe dementia drugs was related to extensive dementia management by primary care physicians when compared with low permissiveness. This could be of interest for dementia care stakeholders as a way to incentivize dementia management [29]. Since studies exploring contemporary dementia management across different countries from a primary care perspective are rare *Unburdening* dementia could be a key for developing policies of dementia management not limited only to health care professionals.

To our knowledge, *Unburdening* presented as a basic social process [15] has not been published before for any health issue, let alone for dementia. Recognising dementia – the first stage of Unburdening – has indeed been much explored [29] and the burden concept often appears in qualitative research on chronic disease management in general and in dementia care in particular [7,8]. Hence, there is a vast literature about caregiver burden in dementia care resulting in emotional distress and burn-out [7]. In a grounded theory dissertation, *unburdening* was a subcore concept explaining caregivers coping by ‘telling their stories’ to the researcher [30].

*Unburdening by drugs* was accompanied by a broadening of guideline indications for dementia drugs. This was in some cases mentioned by physicians as motivated by mercy and equity. In a systematic review of psychosocial factors of patients’ and carers’ experiences of dementia diagnosis and treatment, it was found that dementia drugs gave hope and were considered as ‘worth a try’ although the benefits were not clear [31]. This view of expectations should not be neglected in dementia management to counter therapeutic nihilism [8,29]. Alas, ‘prescription reflex’ doctoring is especially risky for geriatric patients [27]. In this study, physicians thus seemed to rely more on drug treatment to relieve dementia burdens than what is proposed by today’s updated prescription guidelines where deprescribing is actually emphasised in unspecified dementia (but not in Alzheimer’s disease) [17].

It is almost needless to say that *Unburdening by drugs* has general implications for physicians concerning how they deal with many different health issues. We thus propose that *Unburdening by drugs* is what most practicing physicians do every day with or without evidence-based support for the drugs they are prescribing. So why should it be different for patients with dementia?

The conceptual framework of *Unburdening* could be further developed in order to understand practice habits for different diseases and hypotheses based on *Unburdening* may be tested deductively.

## Conclusions

In this grounded theory analysis of an international primary care physician survey, a basic social process that we call *Unburdening* dementia emerged as a resolution for a core concern of the physicians. *Unburdening* is suggested as a tentative explanatory theory of dementia management in primary care starting with *Recognising* a dementia burden by *Identification* and *Assessment* of dementia followed by dementia *Burden Relief*.

Physicians typically identify the burden of both the patient and family members and then assess the cognitive impairment with MMSE in order to relieve the dementia burden, commonly by drug prescriptions, but also by community health and home help services in unburdening tasks. Dementia drug therapy seemed inappropriate in 40% of patients where primary care physicians broadened the guidelines with the purpose of *Unburdening by drugs*. Our findings have implications with regard to how dementia is managed in real world primary care across many jurisdictions.

## Data Availability

All data generated or analysed during this study are included in this published article and its supplementary information files as well as in reference [10]. The datasets analysed during this study will be available from the corresponding author on reasonable request.

## Appendix 1 Appendix 1

### Exploratory survey on dementia diagnosis and treatment in your country (jurisdiction)

**Country………………….……. Name: …… …………… ………. Email:………………@ ………………………**

These questions concern the current situation in your own country or jurisdiction (region or health care district)

Please respond with as many comments as possible since multiple choice questions do not cover all that is important in an exploratory study! If you write your e-mail address we will write you back!

1. Which healthcare professionals are officially responsible for the diagnosis of dementia?
  - Secondary care specialists (neurologist, geriatrician, psychiatrist, neuropsychiatrist)
2. GPs
3. Both

Comments:………….……………………………………………………………………………………………

2.Which are the most popular dementia screening tests used? Please comment if
  - MMSE
Clock Drawing Test
Other. Please specify………………………………………………………………

Comments:………….……………………………………………………………………………………………

3.Are GPs allowed to start prescribing drug treatment for dementia?
  - Yes
No

Comments:………….……………………………………………………………………………………………

4.Is continued dementia drug treatment reimbursed if prescribed by GPs in your country?
  - Yes
No

Comments: ………….………………………………………………………………………………………………………………….

5.Do GPs try to establish a diagnosis of dementia on their own?
  - never
  - rarely
  - often
  - always

Comments: ………….……………………………………………………………………………………………………………

6.Do GPs refer a suspected case of dementia to a secondary care specialist?
  - never
rarely
often
always

Comments: ………….……………………………………………………………………………………………………………

7.What would GPs need to be able to detect dementia better? More than one response option possible.
  - More time in the consultation
Incentives
Easy and short dementia tools
Other………………………………………

Comments:………….……………………………………………………………………………………………

8.Please tell as much as possible about a case of dementia in your practice! (or tell us in a later e-mail)

………….……………………………………………………………………………………………………………

## Appendix 2 Appendix 2

**Exclusion criteria for anti-amyloid trials**

Anti-amyloid trials

Key Exclusion Criteria:

- Any medical or neurological condition (other than Alzheimer's D.) that might be a contributing cause of cognitive impairment.
- Stroke or Transient Ischaemic Attack (TIA) or unexplained loss of consciousness in the past year.
- Clinically significant psychiatric illness in past six months.
- Seizure in the past three years.
- Poorly controlled diabetes mellitus.
- Unstable angina, myocardial infarction, chronic heart failure, or clinical significant conduction abnormalities past year.
- Impaired renal or liver function.
- Human immunodeficiency virus (HIV) infection.
- Significant systematic illness or infection in past 30 days.
- Brain MRI showing acute or sub-acute micro or macrohaemorrhage, >4 microhaemorrhages, cortical infarct or > 1 lunar infarct.
- Any contraindications to brain MRI or PET scans.
- Negative PET scan with any amyloid-targeting ligand last 48 weeks.
- Clinically significant 12-lead electrocardiogram (ECG) abnormalities.
- Alcohol or substance abuse in past 1 year.
- Taking blood thinners (except for aspirin at prophylactic dose or less)
- Changes in medications or doses of medication in past 4 weeks.

Adapted from Sevigny *et al*, Nature 2016 (The antibody aducanumab reduces Aβ plaques in Alzheimer’s disease)

Donepezil Patients were excluded from the donepezil studies if they had insulin-dependent diabetes mellitus or other endocrine disorder, asthma, obstructive pulmonary disease or clinically significant uncontrolled gastrointestinal hepatic or cardiovascular diseases. Patients known to be hypersensitive to cholinesterase inhibitors or who had taken tacrine or other investigational medicines within one month of baseline were excluded. Concomitant medications such as anticholinergics, anticonvulsants, antidepressants and antipsychotics were not allowed. Drugs with central nervous system (CNS) activity were prohibited or partially restricted.

## Appendix 3 Appendix 3

**Exclusion criteria for dementia drug RCTs**

Galantamine Some stable and well-controlled concomitant medical disorders were not reason for exclusion (hypertension, heart failure, diabetes and hypothyroidism). The list of reasons for exclusion was quite extensive and consistent across studies, other neurodegenerative illness, cardiovascular disease, or active cerebrovascular disease, clinically significant infarct dementia, psychiatric, hepatic, renal, pulmonary, metabolic, endocrine, active peptic ulcer, history of epilepsy, drug or alcohol abuse.

Rivastigmine The list of exclusions was not extensive. Patients with severe and unstable illnesses (cardiovascular or pulmonary disease, unstable diabetes mellitus, peptic ulceration within the preceding five years, evidence of alcohol or substance abuse) were excluded, as were subjects taking medications such as anticholinergic drugs, acetyl-choline precursor health food supplements, memory enhancers, insulin and psychotropic drugs.

FACT BOX Donepezil studies Patients were excluded if they had: insulin-dependent diabetes mellitus, other endocrine disorder, asthma, obstructive pulmonary disease or clinically significant uncontrolled gastrointestinal hepatic or cardiovascular diseases. Medications such as anticholinergics, anticonvulsants, antidepressants and antipsychotics. Galantamine studies Patients were excluded if they had: other neurodegenerative illness, cardiovascular disease, or active cerebrovascular disease, clinically significant infarct dementia, psychiatric, hepatic, renal, pulmonary, metabolic, endocrine, active peptic ulcer, history of epilepsy, drug or alcohol abuse. Rivastigmine studies Patients were excluded if they had: severe and unstable illnesses (cardiovascular or pulmonary disease, unstable diabetes mellitus, peptic ulceration within five years, alcohol or substance abuse), medications such as anticholinergic drugs, acetylcholine precursor health food supplements, memory enhancers, insulin and psychotropic drugs. Adapted from: AChEI, Cholinesterase inhibitors for Alzheimer’s disease, Birks J, The Cochrane Library 2009, Issue 1
